# Artificial Neural Network-Based Early-Age Concrete Strength Monitoring Using Dynamic Response Signals

**DOI:** 10.3390/s17061319

**Published:** 2017-06-07

**Authors:** Junkyeong Kim, Chaggil Lee, Seunghee Park

**Affiliations:** 1 Department of Civil & Environmental System Engineering, Sungkyunkwan University 2066, Seobu-ro, Jangan-gu, Suwon-si, 16419 Gyonggi-do, Korea; junk135@nate.com (J.K.); tolck81@gmail.com (C.L.); 2 School of Civil & Architectural Engineering, Sungkyunkwan University 2066, Seobu-ro, Jangan-gu, Suwon-si, 16419 Gyonggi-do, Korea

**Keywords:** early-age concrete strength estimation, artificial neural network, electromechanical impedance, harmonic wave, embedded piezoelectric sensor

## Abstract

Concrete is one of the most common materials used to construct a variety of civil infrastructures. However, since concrete might be susceptible to brittle fracture, it is essential to confirm the strength of concrete at the early-age stage of the curing process to prevent unexpected collapse. To address this issue, this study proposes a novel method to estimate the early-age strength of concrete, by integrating an artificial neural network algorithm with a dynamic response measurement of the concrete material. The dynamic response signals of the concrete, including both electromechanical impedances and guided ultrasonic waves, are obtained from an embedded piezoelectric sensor module. The cross-correlation coefficient of the electromechanical impedance signals and the amplitude of the guided ultrasonic wave signals are selected to quantify the variation in dynamic responses according to the strength of the concrete. Furthermore, an artificial neural network algorithm is used to verify a relationship between the variation in dynamic response signals and concrete strength. The results of an experimental study confirm that the proposed approach can be effectively applied to estimate the strength of concrete material from the early-age stage of the curing process.

## 1. Introduction

Concrete is one of the most common materials used to construct civil infrastructures, such as buildings and bridges. However, it is also one of the most difficult materials to manage because concrete is a mixture, consisting of cement, water, sand, gravel, and other aggregates. Due to its heterogeneous property, it is difficult to mathematically predict the compressive strength of concrete during and/or after curing. In particular, predicting the compressive strength during the curing process is important to reduce the construction time and cost by determining the appropriate curing time to achieve sufficient strength. The in situ strength of concrete structures can be determined with high precision by performing a destructive strength test and material analysis on core samples extracted from the structure [1]. However, this method can lead to destruction of the integrity of the host concrete structure. To overcome this problem, a range of nondestructive testing (NDT) methods has been developed to monitor the strength, without damaging the host structure. These NDT methods can be categorized into two classes: thermal property-based NDT, and mechanical property-based NDT.

Thermal property-based concrete strength NDT methods focus on the hydration heat occurring during the curing process. Concrete hardens due to the hydration process between water and cementitious materials. Because the hydration process involves an exothermic reaction, the concrete generates a certain amount of heat during the curing process. The strength of concrete can be estimated from the degree of hydration, and it can be calculated by measuring the hydration heat. Therefore, the strength of concrete can be estimated by measuring the thermal history of concrete through thermocouples, fiber optic sensors, or other thermal sensors [2,3]. The physical property-based concrete strength estimation methods are based on the change of mechanical properties. Ultrasonic-based methods are general NDT methods used for the early-age monitoring of concrete. The properties of ultrasonic wave propagation, such as velocity or attenuation, are affected by the change of physical properties [4,5,6,7,8]. Thus, the strength of concrete can be monitored by tracking the changes in ultrasonic wave propagation. Also, an electromechanical impedance method using piezoelectric sensors could use to estimate the strength of concrete. The strength of concrete can be estimated by measuring the resonant frequency of impedance [9,10], calculating the RMSD (root mean square deviation) of impedance signals [11], or impedance spectrum analysis [12]. Furthermore, a range of methods based on the acoustical, electrical, magnetic, optical, radiographic, and other mechanical properties of concrete have been studied [13].

Because the concrete is heterogeneous material, the strength of concrete is hard to estimate using a formal equation. To overcome this limitation, the neural network was used to estimate the strength of concrete. The strength of HPC (High Performance Concrete) can be modeled using a modified neural network architecture [14] and fuzzy-ARTMAP neural network by an analysis of the mix proportion [15]. The main benefits in using a neural network are that all of the behavior of a material can be represented within the unified environment of a neural network. Also, the neural network-based model is built directly from experimental data using the learning capabilities of the neural network [15].

In this context, this study proposes a novel method to estimate the strength of concrete at the early-age stage by integrating an artificial neural network algorithm with dynamic response signals of the concrete material. The dynamic response signals of the concrete, including both electromechanical impedances and guided ultrasonic waves, are obtained from an embedded piezoelectric sensor module. The cross-correlation coefficients of the electromechanical impedance signals and the amplitudes of the guided ultrasonic wave signals are selected to quantify the variations in dynamic response signals according to the strength of the concrete. Furthermore, an artificial neural network algorithm is used to verify a relationship between the variations in dynamic response signals and concrete strength. The results of an experimental study confirm that the proposed approach can be effectively applied to estimate the strength of concrete materials from the early-age stage of the curing process.

## 2. Early-Age Concrete Strength Estimation Technique

### 2.1. Embedded Piezoelectric Sensor

Piezoelectric sensors can interconvert mechanical energy and electrical energy. Due to this piezoelectric effect, a piezoelectric sensor can be used simultaneously as both an actuator and a sensor. This study employs a lead zirconate titanate (PZT) patch to generate vibration and waves to the concrete structure, and measure the dynamic responses of the concrete [16,17]. Table 1 shows the size and major properties of the PZT used in this study.

**Figure 1:**
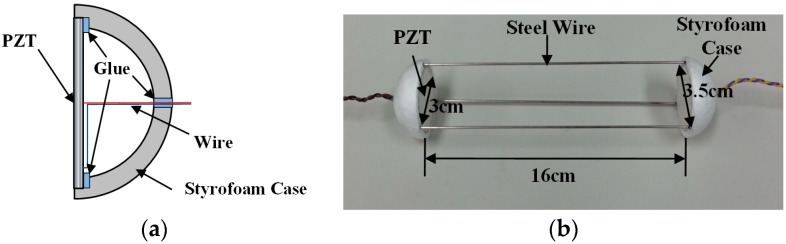
Embedded piezoelectric sensor. (**a**) Schematic of the embedded piezoelectric sensor; (**b**) The proposed embedded piezoelectric sensor module.

To obtain dynamic responses from the inside of concrete structures, the sensors should be embedded within the concrete. However, the PZT can be easily broken by stresses, such as the thermal stress and shrinkage stress of concrete. To protect the PZT in the concrete, an embedded piezoelectric sensor has been developed [18]. The novel embedded piezoelectric sensor is fabricated to improve the signal quality, using a hemi-spherical hollow Styrofoam case, as Figure 1a shows. The embedded piezoelectric sensor allows one side of the PZT to maintain free boundary condition, even though the sensor is embedded within the concrete media. Thus, the embedded piezoelectric sensor can measure the electromechanical impedance as if it were attached to the concrete surface. The electromechanical impedance is measured using a single sensor with a self-sensing technique, and the harmonic wave propagation is measured using two sensors; one is used to generate the harmonic waves, and the other senses the propagated waves. The embedded piezoelectric sensor module consists of two embedded piezoelectric sensors to simultaneously measure the impedance and harmonic wave propagation. Figure 1b shows the size of the module.

### 2.2. Electromechanical Impedance Measurement for Concrete Strength Estimation

**Figure 2:**
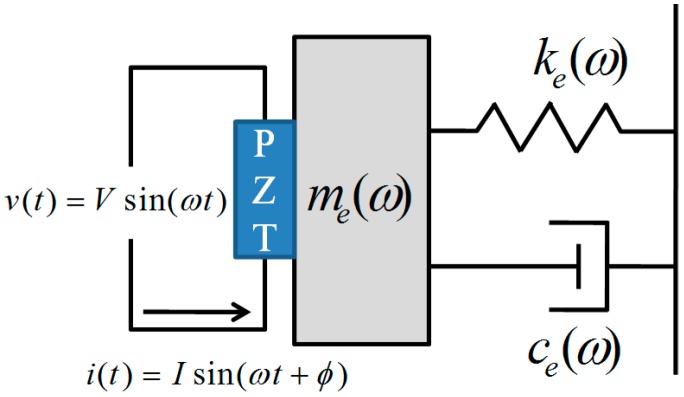
Electromechanical coupling between the lead zirconate titanate (PZT) and the host structure.

The electromechanical impedance (EMI) method has been developed for structural health monitoring, damage detection, and NDT [19,20]. If the PZT is attached to the host structure and an alternating electric voltage is applied to the PZT, the elastic waves generated by the PZT are transmitted to the host structure. The responses on the waves represent the mechanical impedance of the host structure as shown in Figure 2. The structural impedance directly reflects the effective electrical impedance through the mechanical coupling effect between the PZT and host structure. The electromechanical impedance of the PZT, as coupled to the host structure, is given by [16]

(1) $$Z(\omega )=\frac{1}{i\omega C}{\left(1-\kappa_{31}^{2}\frac{k_{str}(\omega )}{k_{PZT}+k_{str}(\omega )}\right)}^{-1}$$

where, Z(ω) is the electromechanical impedance, C is the zero-load capacitance of the PZT, $\kappa_{31}$ is the electromechanical cross coupling coefficient of the PZT, $k_{str}(\omega )$ is the dynamic stiffness of the structure, and $k_{PZT}$ is the stiffness of the PZT.

The dynamic stiffness of concrete changes, according to the development of strength during the curing process. Also, the EMI signals measured in the concrete media should vary during the curing stage. Therefore, the strength of concrete can be estimated by tracking the variation of the EMI signals.

**Figure 3:**
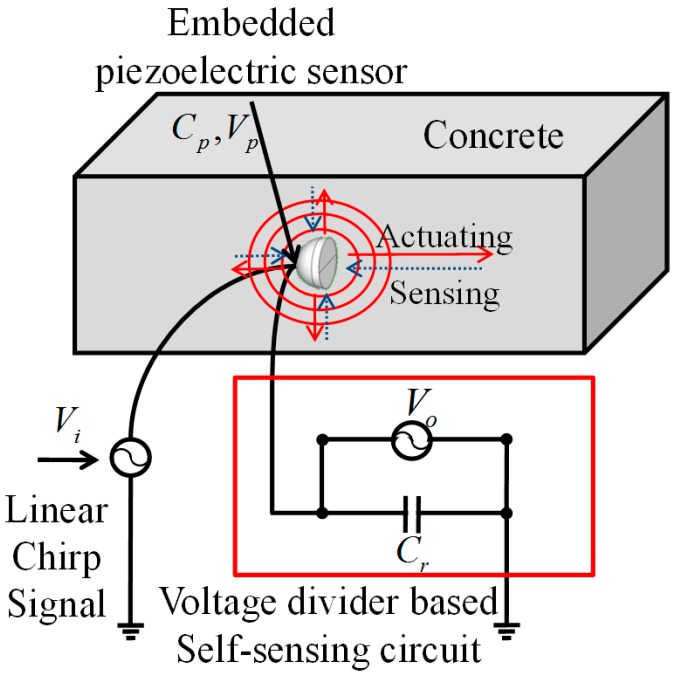
Schematic diagram of self-sensing based impedance measurement.

In this study, an EMI measurement system based on a self-sensing technique with a single embedded piezoelectric sensor was used. A voltage divider-based self-sensing circuit as described in Figure 3 is suitable for use in cast-in-place concrete because it is inexpensive, and has sufficient accuracy to monitor the development of strength, even though the EMI signals are less accurate than when using other impedance measurement methods [21,22].

### 2.3. Harmonic Wave Propagation Measurement for Concrete Strength Estimation

The harmonic wave measurement method is also used to estimate the strength of concrete. The embedded piezoelectric sensor module consists of two embedded piezoelectric sensors, one of which introduces a harmonic wave to the concrete, while the other senses the propagated waves.

**Figure 4:**
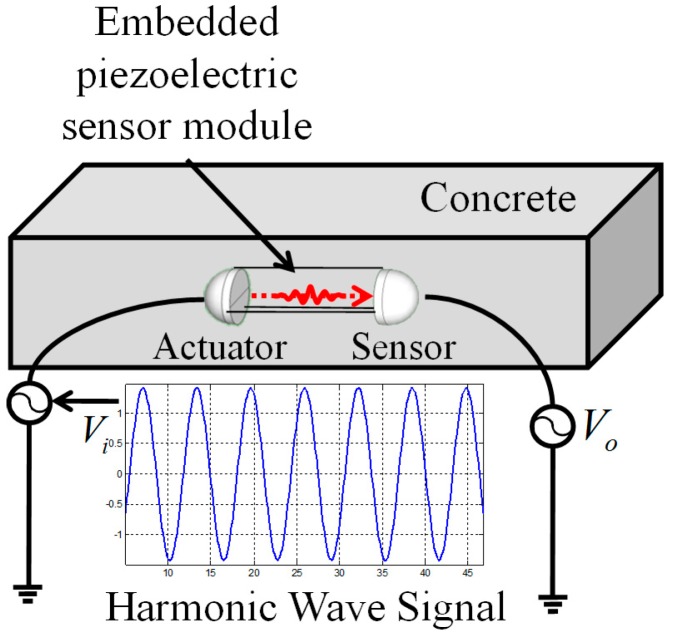
Schematic diagram of harmonic wave measurement.

The harmonic wave propagation in the concrete media can be idealized as one-dimensional longitudinal wave propagation, as Figure 4 shows.

The wave equation of a one-dimensional longitudinal wave is:

(2) ∂2u∂x2=1cb2∂2u∂t2(cb2=Eρ)

where, u is the displacement of an element, and ρ is the density of material. The average power p of the harmonic response over a period can be expressed as:

(3) p=EA2ω22cbEρA2ω22, A=1ω(4p2Eρ)14

where, *A* is the harmonic amplitude, and ω is the angular frequency [23].

Concrete is a cementitious material that has the adhesive and cohesive properties to bond inert aggregates into a solid mass of adequate strength and durability. After casting, the concrete gradually stiffens, until the hydration is completed. The physical properties of concrete, especially the strength, are rapidly changed during curing process. Young’s modulus (*E*) is the factor that affects the most change, not only of the strength of concrete, but also of the amplitude of harmonic wave propagation. Hence, the strength of concrete can be estimated by measuring the amplitude of harmonic wave propagation through concrete [5].

### 2.4. Artificial Neural Network for Concrete Strength Estimation

Concrete is a composite material that consists of cement, water, and aggregates. Because of this heterogeneity, the strength of concrete cannot be clearly derived algebraically by mapping the signal variation and the actual strength. The neural network algorithm, a pattern recognition method, is utilized to deal with the complex properties of concrete [24,25].

The artificial neural network (ANN) comprises a number of processing elements that are connected to form layers of neurons, although the networks may be complex. The missing links between sets of inputs and outputs are found by determining the optimal synaptic weights, based on the available training data of the inputs and outputs [26]. A supervised multi-layer feed-forward ANN with backpropagation is typically employed.

The input to the ANN are the variables, ($x_{1}$, $x_{2}$, … , $x_{N}$), which are weighted by $w_{j,i}^{h}$ and bias $b_{j}^{h}$; and the output results, ($y_{1}$,$y_{1}$, … ,$y_{k}$), feed the hidden layers. The output of the j-th hidden unit can be described as:

(4) $$y_{j}^{h}=F_{1}\left(b_{j}^{h}+\sum_{i=1}^{N}w_{j,i}^{h}\cdot x_{i}\right)$$

where, h refers to the quantities of the hidden layer, and $F_{1}$ is a sigmoid nonlinear function. The output of the ANN is biased and weighted by the sum of the hidden layer outputs:

(5) $$y_{l}=F_{2}\left(b_{l}^{0}+\sum_{j=1}^{H}w_{j,i}^{0}\cdot y_{j}^{h}\right)$$

where, o refers to the output unit, H refers to the number of units in the hidden layer, and $F_{2}$ refers to a linear activation transfer function. The ANN is trained starting with a random set of weights and biases, and the output is calculated for every input data. Then, the error is calculated from the output layer and propagated backwards to modify the hidden layers, and has historically been called the backpropagated error. This learning algorithm is called backpropagation [27]. The training of the network involves a set of inputs for which the specified output is known. The training process is finished when the error is smaller than a desired value, or the process reaches a maximum specified number of iterations [28].

To build the ANN for concrete strength estimation, the network is trained using the set of inputs representing the condition of the concrete, and the output that is measured compressive strength for each set of conditions. The inputs are the dynamic response features extracted from impedance/wave measurements. After training, the strength of concrete can be estimated by inputting the measured data set into the trained ANN.

## 3. Experimental Study

### 3.1. Experimental Setup and Test Procedure

**Figure 5:**
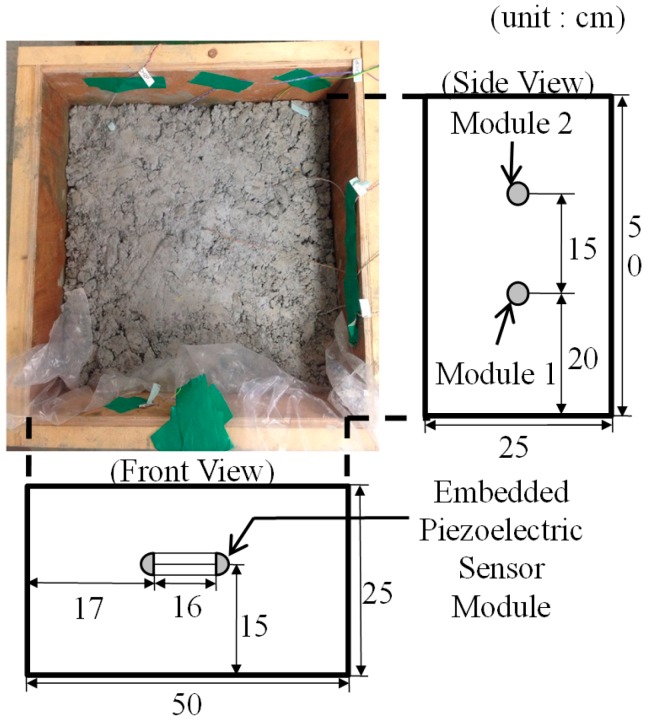
Geometric information of the test specimen.

An experimental study was performed to verify the proposed strength estimation method. A 50 × 50 × 25 cm concrete specimen was cast, and two piezoelectric sensor modules were embedded during the casting process, as Figure 5 shows. Module 1 was used to obtain dynamic response data sets for training the ANN, and module 2 was used to verify the trained ANN. Table 2 shows the mix proportion of the concrete.

The specimen was cured in air at room temperature. The reference strength of the specimen was measured at the early-age of the curing stage from a destructive test using a Universal Test Machine (UTM), with Φ10 × 20 cm standard cylinders cast with the same concrete as that of the test specimen, at 16, 25, 38, 51, 75, and 99 h after casting. Table 3 shows the strength of concrete at the early-age of the curing stage from destructive testing.

The EMI and harmonic wave signals were measured using the NI-PXI DAQ system (1042Q), and a self-sensing circuit board was used for EMI measurement. The EMI was measured in the frequency range of 5 kHz~200 kHz, with a 2 MHz sampling rate. The harmonic wave was actuated with a 100 kHz frequency signal, and measured with a 5 MHz sampling rate. The dynamic response signals were measured every hour after casting, up to 100 h.

### 3.2. Result of EMI Measurement

**Figure 6:**
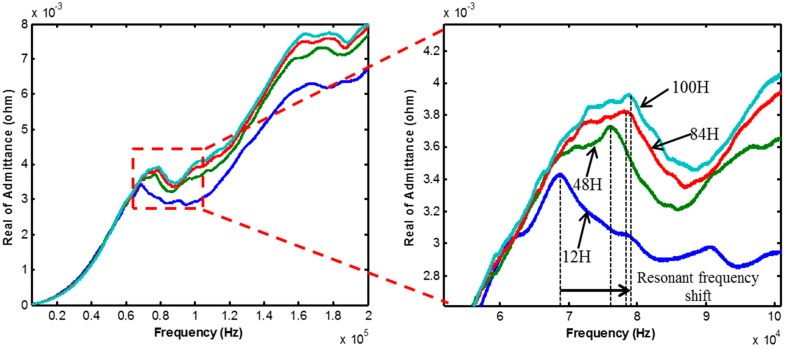
Electromechanical impedance (EMI) variation of module 1.

**Figure 7:**
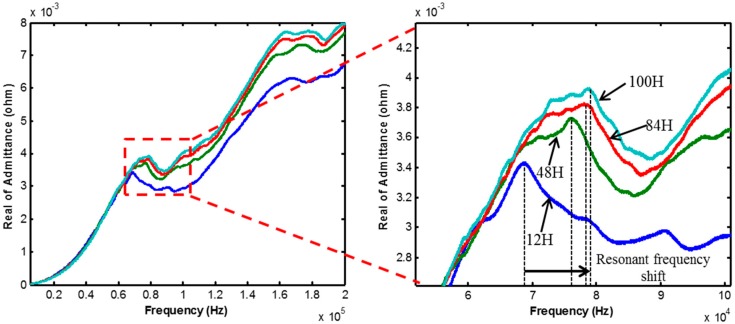
EMI variation of module 2.

The measurement result of EMI is shown below. Figure 6 shows the results measured by module 1, and Figure 7 shows the result measured by module 2 at curing ages of 12, 48, 84, and 100 h. It is observed that both the EMI signals change during the early-age of the curing stage. In particular, the resonant frequency (the frequency at the peak of the signal) gradually increases during the curing age. Because the resonant frequency is proportional to the stiffness, it should increase with time.

The cross-correlation coefficient index (1-*CC*) was calculated to provide quantitative information about signal variation. The 1-*CC* values were derived using the following equation:

(6) 1−CC=1−1N−1∑i=1N(Re(Z0)−Re(Z0¯))(Re(Zi)−Re(Zi¯))σZ0σZi

where, Re(Z_0_) is the real part of the impedance function at the baseline (the EMI data before embedment), Re(Z_i_) is the real part of the impedance of the *i*-th hour at each measured data, Re(Z0¯) and Re(Zi¯) are the average of each data set, $\sigma_{Z_{0}}$ and $\sigma_{Z_{0}}$ are standard deviation of each dataset and *N* is the total number of datasets.

**Figure 8:**
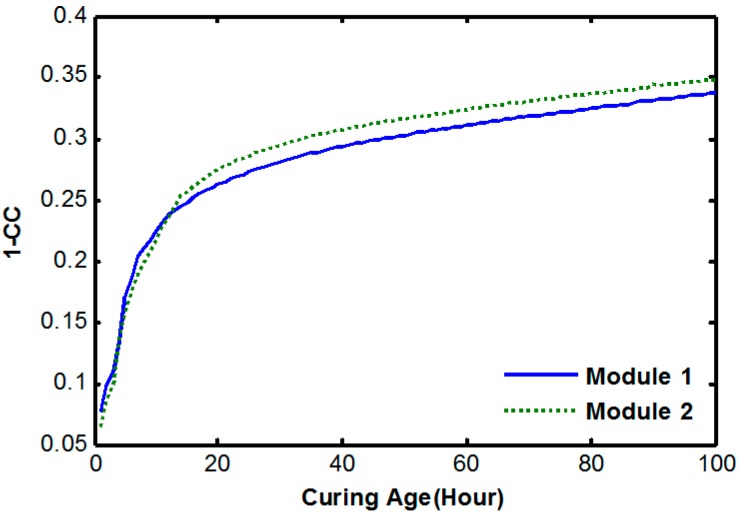
Cross correlation variations during the early-age of the curing stage.

Figure 8 reveals that the 1-*CC* data of modules 1 and 2 show almost the same pattern, which has a similar trend to the typical strength curve at the early-age of the curing stage.

The equation for estimating the strength was derived using a linear regression method using the 1-*CC* of the impedance signal. The estimation equation from linear regression is shown in Equation (7).

(7) S(MPa)=210.5×CC−30.24

**Figure 9:**
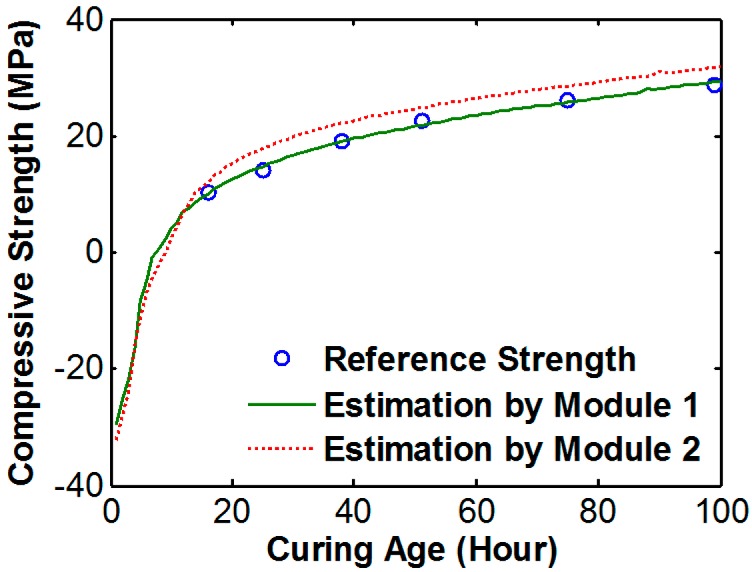
Strength estimation result using the regression model.

Figure 9 shows the result of the strength estimation using that equation during 100 h. The estimation result after 20 h shows almost the same value as the reference strength. However the estimation results before 20 h are decreased very steeply, and the estimation strength before 5 h represents a minus value.

### 3.3. Result of Harmonic Wave Measurement

**Figure 10:**
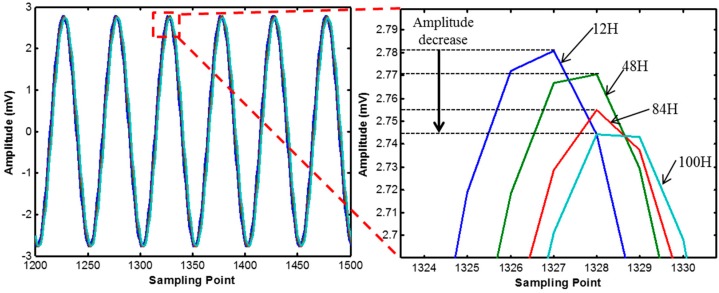
Harmonic wave signal variation of module 1.

**Figure 11:**
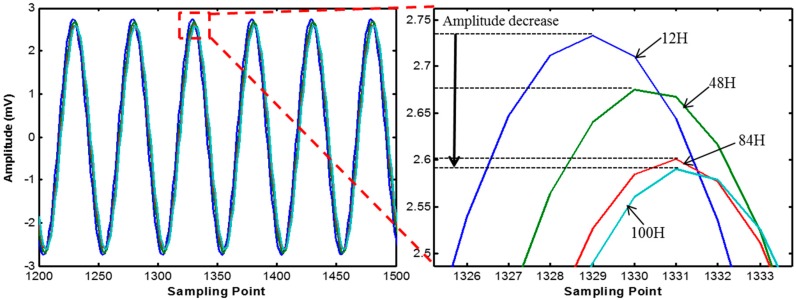
Harmonic wave signal variation of module 2.

Figure 10 and Figure 11 show the variations of harmonic wave signals measured by module 1 and 2 due to the curing process. As the curing age increases, the amplitude of the internal harmonic wave decreases.

**Figure 12:**
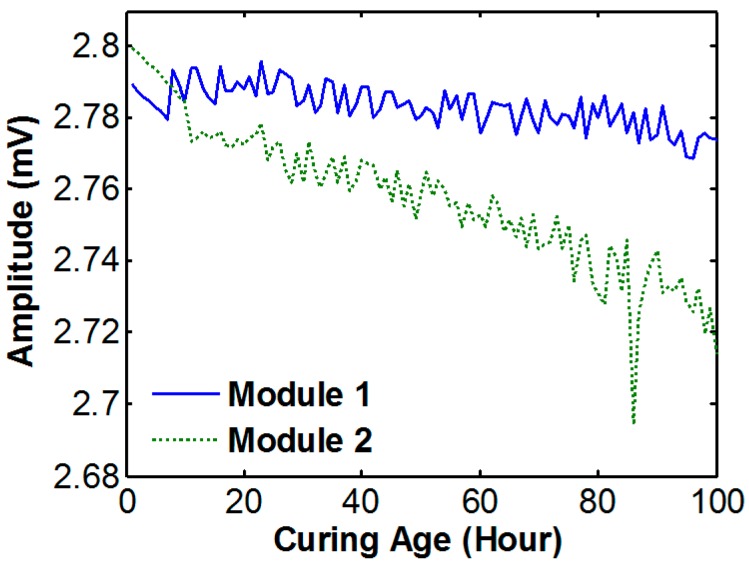
Wave propagation amplitude change during the early-age of the curing stage.

To quantify the signal variation, the amplitude of the harmonic wave is extracted as a feature of wave propagation. Figure 12 shows the amplitude changes of the two modules.

Although the slope of decreasing amplitude is not clear and both data are not the same, the two sets of amplitude data show that the amplitude decreases according to the increase of strength. This is caused by the inverse relationship between the wave amplitude, and the Young’s modulus (E) of the propagation media. However the amplitudes only change by 1%, and the variation patterns are dissimilar between module 1 and 2. This is due to the fact that the path of wave was different because the placement of aggregates. Thus the amplitude of harmonic wave is not recommended to estimate concrete strength estimation in this specimen.

### 3.4. ANN-Based Concrete Strength Estimation

The features of the dynamic response signals have a certain pattern related to the increase in concrete strength. The ANN was utilized to recognize the pattern and estimate the strength of concrete, using the features from the dynamic response signals. The transfer function of ANN was tan-sigmoid function and the learning rule was Levenberg–Marquadt backpropagation. Also, the ANN had 10 hidden layers and one output layer.

The input data consisted 100 sets of the 1-*CC* of the impedance signals and the output data was the reference strength. The unmeasured reference strength was calculated by the interpolation using the measured reference strength and the curing time. The ANN was trained by data from module 1 and the trained ANN was verified by inputting the data from module 2.

**Figure 13:**
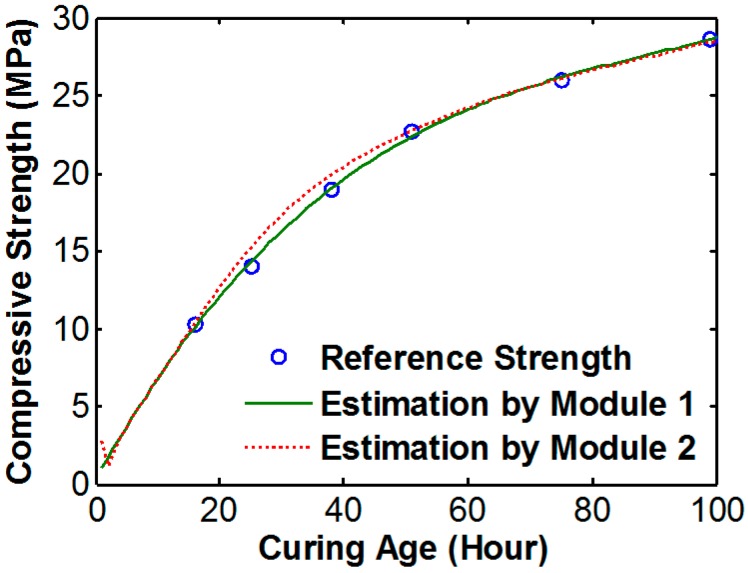
Strength estimation result using the artificial neural network (ANN).

Figure 13 shows the result of concrete strength estimation using the ANN model. The maximum error between the reference strength and estimation strength was 1.33 MPa. Also, the strength before 16 h was reliably estimated. Because the neural network could connect the relationship between signal variation and strength with higher order than linear regression (and also the network trained using all of data from module 1), the estimation result from ANN is robust against error than linear regression results. According to the result, the proposed ANN model could estimate the strength of the test specimen with negligible error.

## 4. Conclusions

In this paper, an ANN model trained by dynamic response signals is proposed to estimate early-age strength monitoring of concrete. The dynamic response signals, the electromechanical impedance and ultrasonic harmonic waves are changed by the strength variation of the host concrete. The EMI and harmonic waves were measured during the curing process using two embedded piezoelectric sensor modules, and the reference strength was measured using a destructive test on standard cylinders. From a series of experimental studies, it was confirmed that the dynamic response signals obtained from the concrete during the early-age of the curing process has a pattern according to the strength. According to the strength gain, the resonant frequency of EMI was gradually increased and the amplitude of harmonic wave was decreased. The variation in resonant frequency of the two sensor modules had a similar tendency while the variation in amplitude of the harmonic wave response of the two sensor modules had a dissimilar result caused by the path difference. Thus, the EMI variation was selected to estimate the strength of concrete. The cross-correlation coefficient was used to quantify the variation in EMI according to the strength gain. Furthermore, an artificial neural network algorithm was used to define the relationship between the variation in EMI and the strength of concrete. The ANN model was trained by the variation in 1-CC of EMI measured by sensor module 1 and verified by the data of sensor module 2. The results conclusively confirmed that the proposed approach could be effectively applied to estimate the strength of concrete material from the early-age stage of the curing process.

## Figures and Tables

**Table 1:** PZT material properties.

APC 850 WFB Series					
Size (mm)		Electromechanical Coupling Factor		Piezoelectric Charge Constant ($10^{-12}$ m/V)	
**Diameter**	**Thickness**	$\kappa_{33}$	$\kappa_{31}$	$d_{33}$	$d_{31}$
30.00	0.508	0.72	0.36	400	−175

**Table 2:** Mix proportion of test concrete.

Water (kg/m^3^)	Cement (kg/m^3^)	Silica Fume (kg/m^3^)	Sand (kg/m^3^)	Gavel (kg/m^3^)	AE (%)
155	433	22.8	737	941	0.45

**Table 3:** Reference strength of the test specimen.

Curing Age (h)	16	25	38	51	75	99
Compressive Strength (MPa)	10.24	14.06	18.95	22.65	26.02	28.63
